# A functional role for the colleters of coffee flowers

**DOI:** 10.1093/aobpla/plt029

**Published:** 2013-06-28

**Authors:** Juliana Lischka Sampaio Mayer, Sandra Maria Carmello-Guerreiro, Paulo Mazzafera

**Affiliations:** Departamento de Biologia Vegetal, Instituto de Biologia, CP 6109, Universidade Estadual de Campinas, 13083-970 Campinas, SP, Brazil

**Keywords:** Alkaloids, caffeine, *Coffea arabica*, colleter, histochemistry, scanning electron microscopy.

## Abstract

Colleters are protuberances or trichomes that produce and release an exudate that overlays vegetative or reproductive buds. Colleters have a functional definition, as they are thought to protect young tissues against dehydration and pest attack. Decaffeinated coffee plants, named Decaffito^®^, have recently been obtained through chemical mutagenesis, and in addition to the absence of the alkaloid, the flowers of these plants open precociously. Decaffito mutants exhibit minimal production and secretion of the exudate by the colleters. We compared these mutants with normal coffee plants to infer the functional role of colleters and the secreted exudate covering flower buds. Decaffito mutants were obtained by sodium azide mutagenesis of *Coffea arabica* cv. Catuaí seeds. Wild-type plants were used as controls and are referred to as Catuaí. The flower colleters were analysed by scanning and transmission electron microscopy in addition to histochemical analysis. Histochemical analysis indicated the presence of heterogeneous exudate in the secretory cells of the colleters of both variants of coffee trees. Alkaloids were detected in Catuaí but not in Decaffito. Transmission electron microscopy revealed that the secretory cells in the Catuaí colleters possessed the normal and common characteristics found in secretory structures. In the secretory cells of the Decaffito colleters, it was not possible to identify any organelles or even the nucleus, but the cells had a darkened central cytoplasm, indicating that the secretion is produced in low amounts but not released. Our results offer a proof of concept of colleters in coffee, strongly indicating that the exudate covering the flower parts works as an adhesive to keep the petals together and the flower closed, which in part helps to avoid dehydration. Additionally, the exudate itself helps to prevent water loss from the epidermal cells of the petals.

## Abstract (precis)

Colleters have functional definitions such as protection against dehydration, and pathogens and insects attack. So far this definition is intuitive and no direct proof has been provided. We compared flowers of coffee mutants (Decafitto), which exhibit minimal production and secretion of exudate by colleters, with normal plants, to provide a proof of concept that the exudate covering the flowers plays a role against dehydration and acts as an adhesive to keep the petals united until flower opening.

## Introduction

Colleters have been defined as secretory structures present in different organs of members of >60 angiosperm families, including Rubiaceae, Loganiaceae and Apocynaceae (Thomas and Dave 1990; Miguel *et al*. 2009). Structurally, they can appear as trichomes or as emergences that are formed from both epidermal and subepidermal tissues (Foster 1949; Appezzato-da-Gloria and Estelita 2000; Evert 2007).

Morphology, location and the chemical nature of the exudate are the criteria used to define the term colleter, but in fact the functional concept is the common link connecting most reports of this structure (Mayer *et al*. 2011). The term colleter originates from the Greek word ‘colla’, which means glue (Foster 1949). The nature of the sticky resinous or mucilaginous substance released by the colleters is diverse, and polysaccharides, proteins and lipids have been described as components (Miguel *et al*. 2006). These structures differentiate early, and their function seems to be to provide physical or chemical defences for the shoot apex and lateral buds against insect and pathogen attack. However, the occurrence of colleters is not limited to these plant parts, as they also occur in reproductive organs and seedlings (De-Paula and Oliveira 2007; Mayer *et al*. 2011). Other reports suggest that colleters and the exudate protect juvenile plant structures against dehydration because the exudate permeates and overlays the entire meristem and juvenile organs (Thomas and Dave 1989; Thomas *et al*. 1989; Klein *et al*. 2004). Appezzato-da-Gloria and Estelita (2000) argued that the exudate prevents water loss in hot tropical climates. In addition to dehydration protection, it has also been suggested that by covering the shoot apex meristem, the exudate may act as a physical barrier (Miguel *et al*. 2010). It is important to supplement morphological, structural and developmental data with information on the secretory product from histochemical tests or chemical analysis to construct an association between the definition of colleter and its functionality (Radford *et al*. 1974).

Although the alleged role of the colleter and its exudate is to protect plant parts against water loss and attack by insects and pathogens, only indirect evidence of this role has been obtained. Only a few reports have analysed the composition of the resinous material and have suggested such a role (Miguel *et al*. 2006 and references therein). According to Miguel *et al*. (2006), who demonstrated an *in vitro* fungicide property (spore germination) of the exudate from the colleters of *Bathysa nicholsonii* K. Schum. (Rubiaceae), the exudate protects the shoot apical meristem against pathogen attack. Another function attributed to the exudate from colleters is related to nutritional aspects regarding bacterial leaf nodule symbiosis with the Rubiaceae species (Horner and Lersten 1968; Lersten 1975).

The colleters in the stipules of *Coffea arabica* (Patel and Zaveri 1975) are classified as ‘standard type’ (Lersten 1974*a*, *b*). This type of colleter is formed by a secretory epidermis and the central axis of parenchyma cells, without vascular tissue. The origin of this type of colleter involves the protoderm and the ground meristem, as described for the colleters of *Caryocar brasiliense* Camb. (Paiva and Machado 2006*a*).

Under Brazilian climate conditions, coffee (*C. arabica*) floral buds start to differentiate from axillary buds in January at the leaf axils that pre-formed in August of the previous year (Majerowicz and Söndahl 2005). During the shorter days of April, the induction of the existing leaf buds to flower buds intensifies. Once they have developed into mature buds, they become dormant. Dormancy coincides with the start of the dry season, and as soon as the first rains of spring begin, flowering is triggered and anthesis occurs (Camargo and Camargo 2001). During development, coffee buds/flowers are covered by a viscous exudate of unknown composition. The functional role of this secretion has never been proved, but it is argued that it protects the dormant coffee floral bud from dehydration during the dry season, as has been suggested for many other plant species.

**Figure 1.**
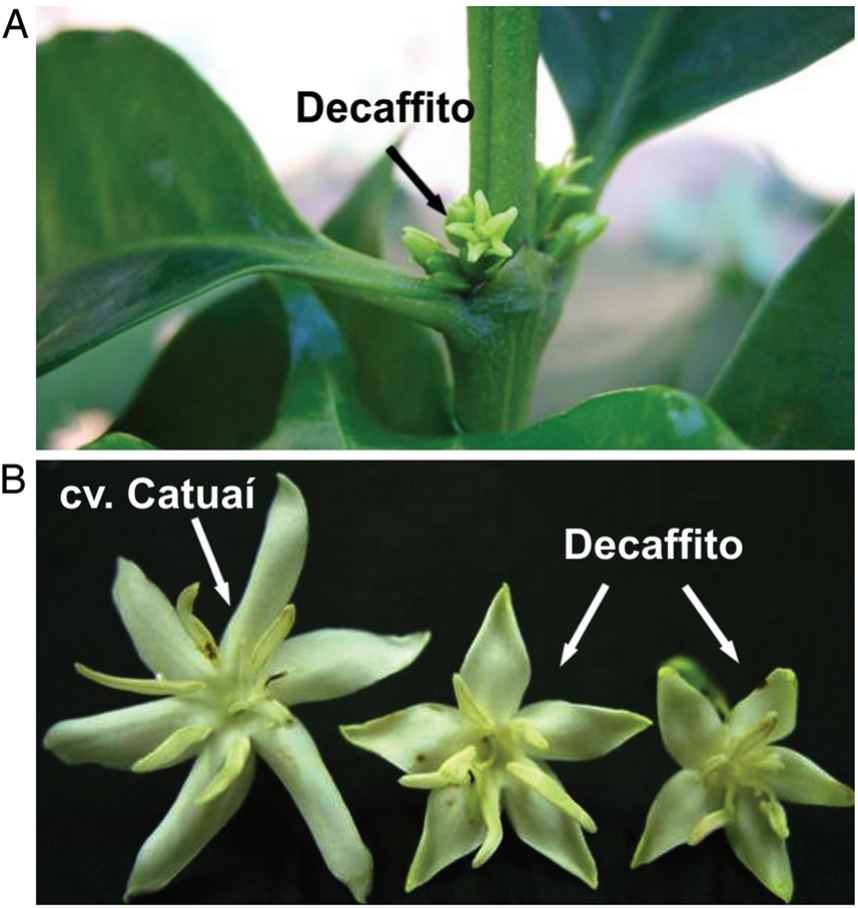
(A) Flowers of Decaffito on a branch of a plant in the field and (B) open flowers of Catuaí and Decaffito.

Recently, we used sodium azide to mutagenize coffee seeds, aiming to obtain decaffeinated coffee plants (P. Mazzafera, unpubl. res.; Borrell 2012). Among the ∼33 000 seedlings analysed, seven were found not to contain caffeine (1,3,7-trimethylxanthine) due to a blockade in the methylation of theobromine (3,7-dimethylxanthine) mediated by caffeine synthase (Mazzafera *et al.* 1994). These plants were grown in the field for 2 years until they blossomed, at which point it was observed that, in addition to lacking caffeine, they exhibited precocious flower opening (see Fig. 1A). The term anthesis will not be used here because we understand that what happens in Decaffito is not a normal process. Although this process happens very early during bud development, the buds curiously also undergo a period of dormancy, i.e. they stop growing during the dry season and start to swell and increase in size with the first rains of spring. The flowers are smaller than normal flowers (Fig. 1B), but they produce viable pollen. By crossing these mutants with caffeinated plants, the descendants recover the normal caffeine content. Normal *C. arabica* plants have cleistogamy, and therefore self-pollination is high (Carvalho 1988), an advantage lost by the decaffeinated mutants as they are more prone to cross-pollination. Controlled crosses showed that every time the decaffeinated mutant was obtained, this early flower opening phenotype was displayed, suggesting a strong genetic link. These mutants were named Decaffito (Mazzafera *et al*. 2009).

Here, we report that the flowers of Decaffito open precociously because of the lack of exudate released by colleters. In normal flowers, the viscosity of the exudate seems to hold the petals together, acting as an adhesive, and does not allow them to open until they absorb water, swell and can then overcome the barrier imposed by the exudate. Furthermore, the exudate seems to protect against dehydration through the formation of a thick layer on the young flower buds, which have numerous stomata on the external petal surface. This information is the first direct evidence for a functional role of colleters and their exudate.

## Methods

### Botanical materials

*Coffea arabica* cv. Catuaí Vermelho and cv. Decaffito were used in this study. The plants were grown in the experimental field of the Department of Plant Biology of the State University of Campinas, Campinas-SP, Brazil. Catuaí is a commercial cultivar and contains ∼1.2 % caffeine on a dry weight (DW) basis in its fruits (Guerreiro Filho and Mazzafera 2003) and 0.8 % DW in its leaves (Guerreiro Filho and Mazzafera 2000).

Decaffito mutants were obtained by treating Catuaí Vermelho seeds with sodium azide (0.003 or 0.01 % in 200 mM sodium phosphate pH 3, 48 h) and then germinating them in a sand bed. Approximately 33 000 plants were analysed for caffeine in the leaves using high-performance liquid chromatography, and seven were found to be almost devoid of caffeine. These plants were then transferred to field conditions (P. Mazzafera, unpubl. res.; Borrell 2012). Samples of the flower buds and flowers at different developmental stages were collected from these plants and used in our studies.

### Light microscopy

Samples were fixed under vacuum as described by Karnovsky (1965; modified by preparation in phosphate buffer pH 7.2) for 24 h and dehydrated in an ethanol series (10, 30, 50 and 70 %) and then in a *tert*-butyl alcohol (TBA) series (70, 85, 95 and 100 %) (Johansen 1940) for 48 h in each solution. The last dehydration in 100 % TBA was repeated three times. A three-fourths volume of solid Paraplast X-tra^®^ (Fisher) was added to the samples in 100 % TBA, and the mixture was maintained at 58 °C. The Paraplast was changed three times, every 12 h. The samples were placed on moulds to solidify, and serial sections (5µm thick) were cut on a rotary microtome (Leica) and distended in heated plates at 48 °C. The Paraplast was removed by immersion of the slides in xylene, and the sections were subsequently rehydrated in absolute ethanol followed by distilled water. The sections were stained with safranin and astra-blue (Gerlach 1969) and mounted in Entellan^®^ synthetic resin (Merck). Photomicrographs were taken with an Olympus BX 51 photomicroscope equipped with an Olympus DP71 camera.

### Histochemistry

Samples were fixed, dehydrated and embedded as described above. The chemical nature of the substances found in the secretory cells of the colleters and the exudate was determined using the following histochemical tests: periodic acid–Schiff's reaction for 1,2-glycol groups present in polysaccharides (McManus 1948); ruthenium red for acid polysaccharides and pectic substances (Johansen 1940); Wagner's reagent for alkaloids (Wagner *et al*. 1984); aniline blue black (Fisher 1968) to identify proteins; and Sudan black B (Pearse 1985) and Nile blue (Cain 1947) for neutral (stained pink) and acidic (stained blue) lipids to identify the aliphatic compounds. Standard control procedures were performed simultaneously.

### Scanning electron microscopy

Samples were fixed as described by Karnovsky (1965; modified by preparation in phosphate buffer pH 7.2) for 24 h, dehydrated in a graded ethanol series and subjected to critical point drying with CO_2_ (Horridge and Tamm 1969). The samples were attached to aluminium stubs and coated with gold (30–40 nm). Finally, the samples were examined under a LEO model VP 435 scanning electron microscope at 20 kV.

### Transmission electron microscopy

Samples of bract with colleters were fixed by Karnovsky's method (Karnovsky 1965; modified by preparation in phosphate buffer pH 7.2), post-fixed in 1 % osmium tetroxide (0.1 M phosphate buffer pH 7.2) for 2 h, dehydrated by an acetone series and embedded in Araldite resin (Roland 1978; Silva and Machado 1999). Ultrathin sections were contrasted with uranyl acetate and lead citrate (Roland 1978) and examined under a Philips EM 100 transmission electron microscope at 60 kV.

## Results

### Morphology of the bud flower

**Figure 2.**
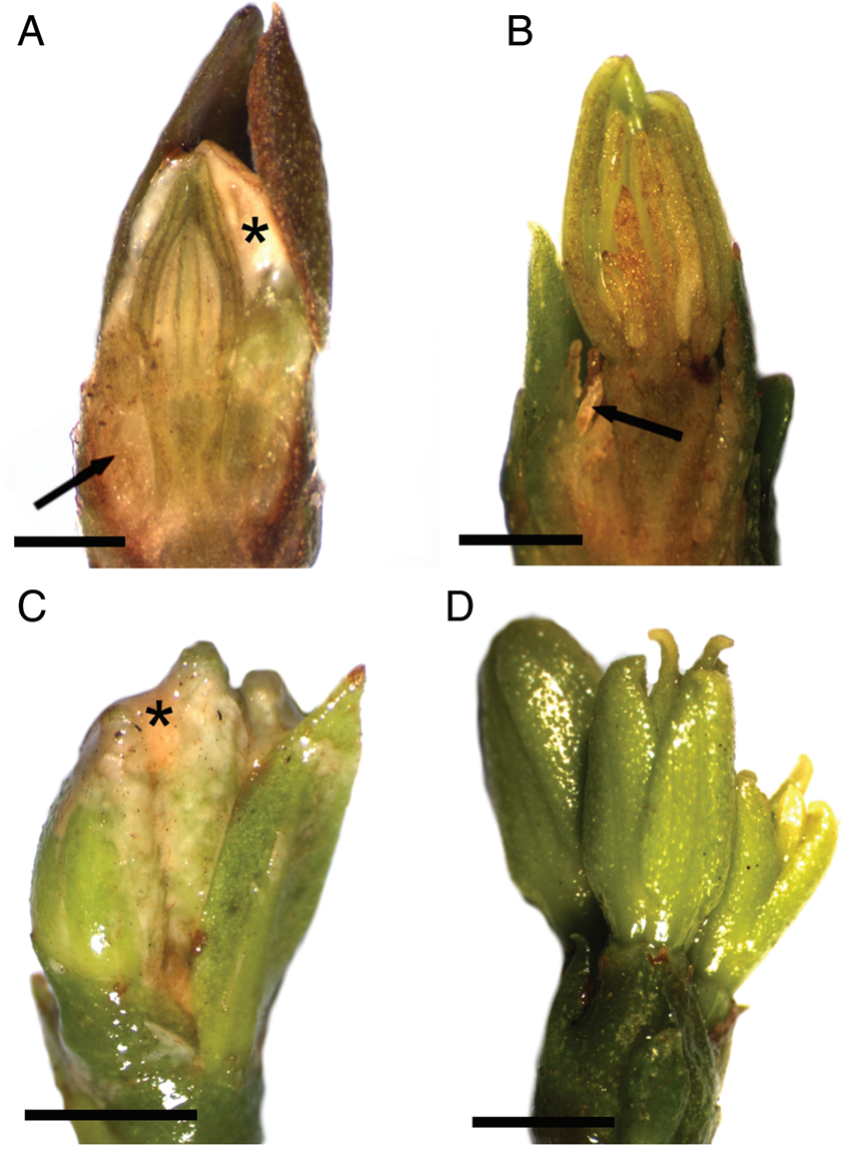
Flower buds of (A and C) Catuaí and (B and D) Decaffito. (A and B) Note the colleters (arrows) and the exudate covering the petals (*). Scale bars: A and B = 1 mm; C and D = 2 mm.

**Figure 3.**
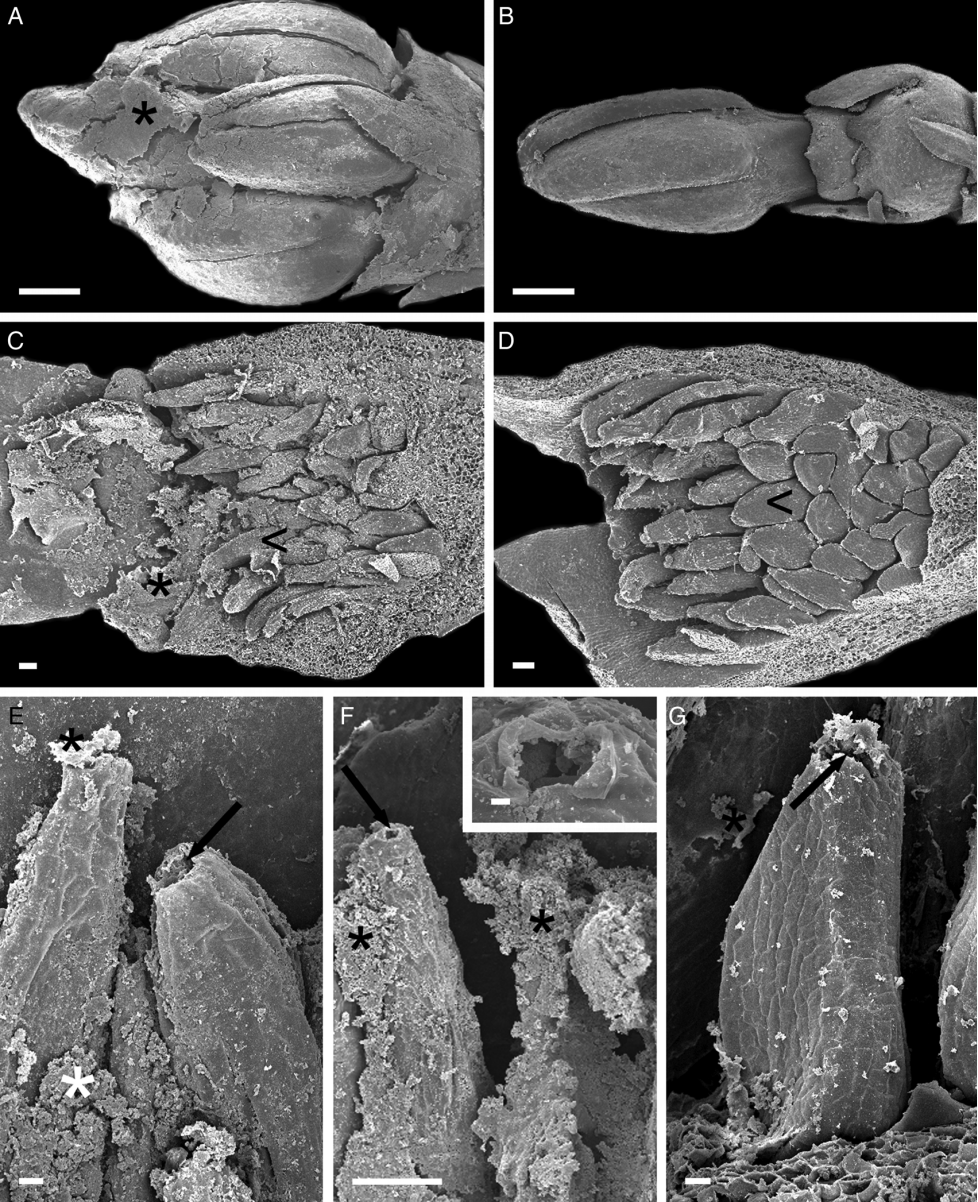
Scanning electron micrographs of flower buds and colleters. (A) Overview of the flower bud of Catuaí: observe the exudate covering the petals; (B) overview of the flower bud of Decaffito; (C and D) overview of colleters (arrowhead) in the bract; (C) Catuaí; (D) Decaffito; (E–G) details of colleters: note the cuticular rupture in the apical portion of the colleter (arrows), exposing the secretory cells and releasing the exudate (*); (E and F) Catuaí: note the apical portion of the colleter in detail; (G) Decaffito. Scale bars: A and B = 1 mm; C, D and F = 100 µm; E and G = 20 µm; F (inset) = 3 µm.

**Figure 4.**
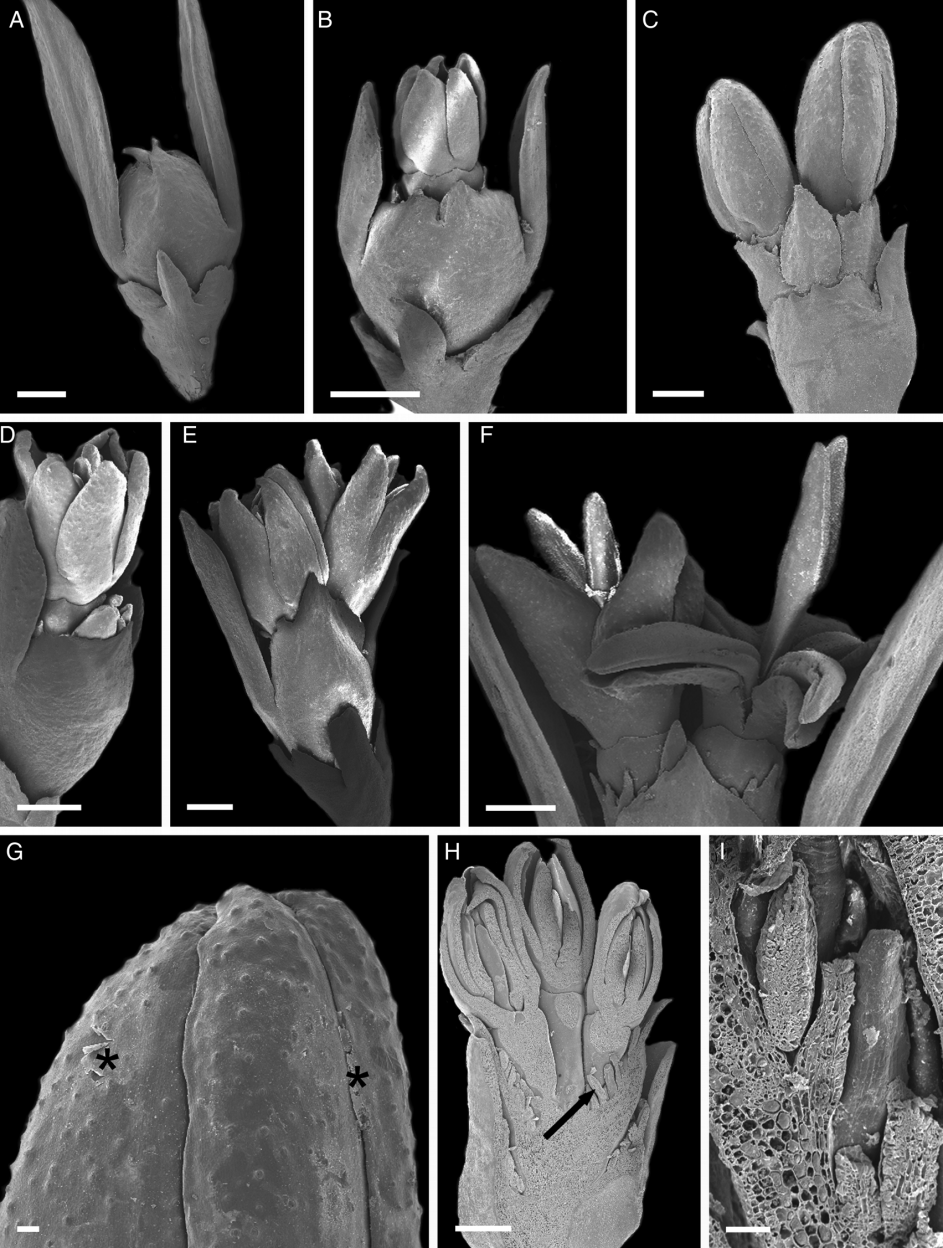
Scanning electron micrographs of the flowers of Decaffito. (A–F) Different stages of the flower bud; note the scarce presence of exudate; (D–F) flower in precocious opening; (G) note the scarce exudate (*) covering the petals; (H) overview of a flower bud, view of the colleter position (arrows) in the adaxial side of the bract; (I) details of colleters. Scale bars: A–F = 1 µm; G and I = 100 µm; H = 1 mm.

**Figure 5.**
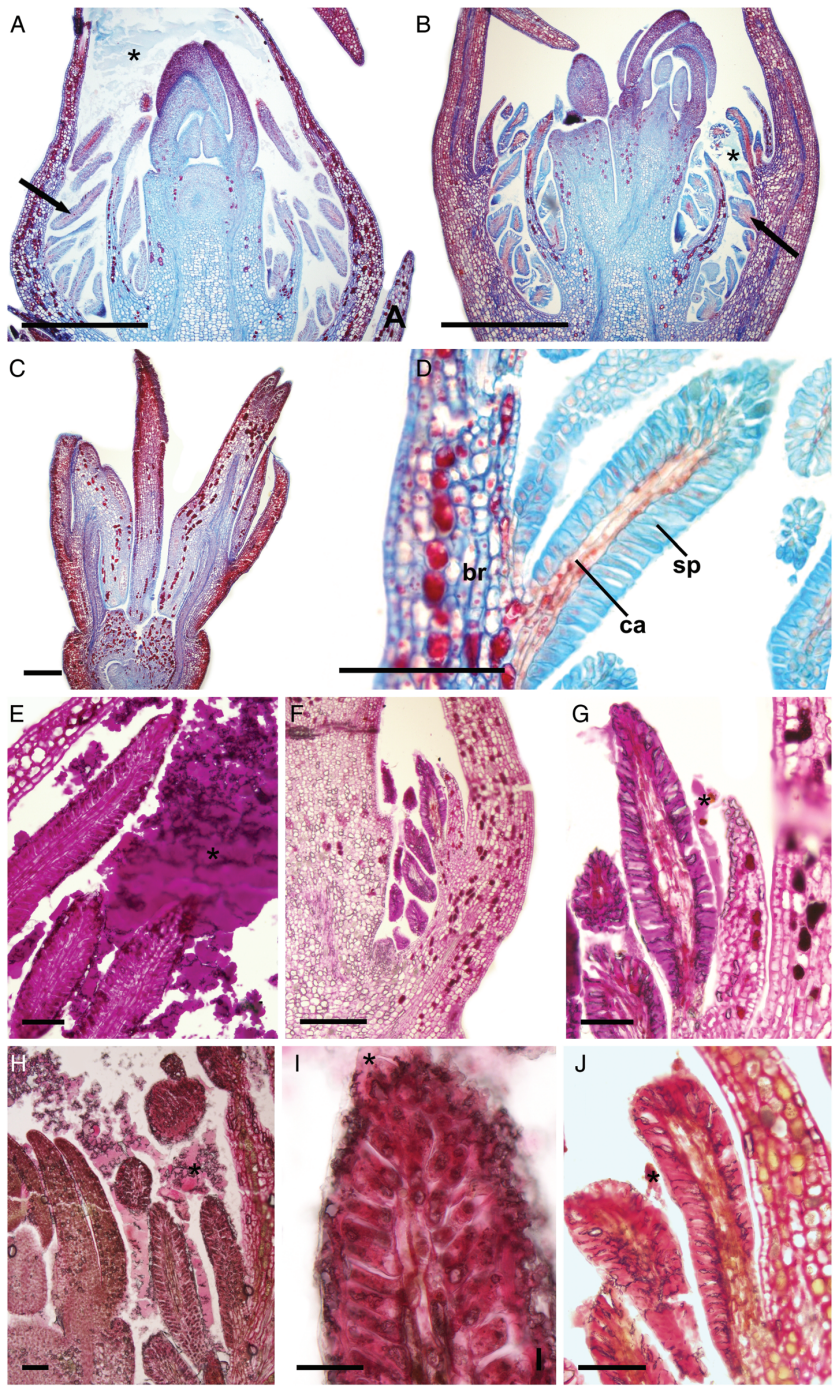
Longitudinal sections of flower buds. (A, E, H and I) Catuaí; (B–D, F, G and J) Decaffito. (A and B) Flower bud at the beginning of development; note the extracellular exudate (*) and colleter (arrows); (C) flower in precocious opening, without complete development of the floral organs; (D) details of the colleter with the secretory palisade epidermis (sp) and the central axis formed by non-secretory parenchyma cells (ca) in the adaxial side of the bract (br); (E–J) histochemical characterization of the exudates; (E–G) periodic acid–Schiff reaction; (H–J) ruthenium red. Scale bars: A and B = 500 µm; C and F = 200 µm; D = 100 µm; E, G, H and J = 50 µm; I = 20 µm.

The Catuaí flower buds had a whitish, thick, viscous exudate covering the petals at different developmental stages before flower opening (Figs 2A, C and 3A). Although the exudate was not visible in fresh samples of Decaffito flowers (Figs 1A and 2B, D), analysis by scanning electron microscopy revealed its presence in minimal amounts (Figs 3B and 4G) when compared with Catuaí. The lack of exudate in Decaffito was observed from the beginning of the development of the reproductive meristem until the precocious opening (Figs 1A, B, 4A–F and 5B, C).

The structures responsible for the secretion of the exudates are the colleters, which are positioned on the adaxial side of the bracts adjacent to the flower buds, as seen for Catuaí (Figs 2A, 3C and 5A, F) and Decaffito (Figs 2B, 3D, 4H, I and 5B–D). The colleters are long, have a short peduncle (Figs 3E–G and 4I) and are formed by a secretory palisade-like epidermis and an axis of non-secretory parenchyma central cells (Fig. 5D). The secretory phase of colleters begins at the induction of the reproductive meristem and remains active during the development of the flower bud.

### Histochemistry

**Figure 6.**
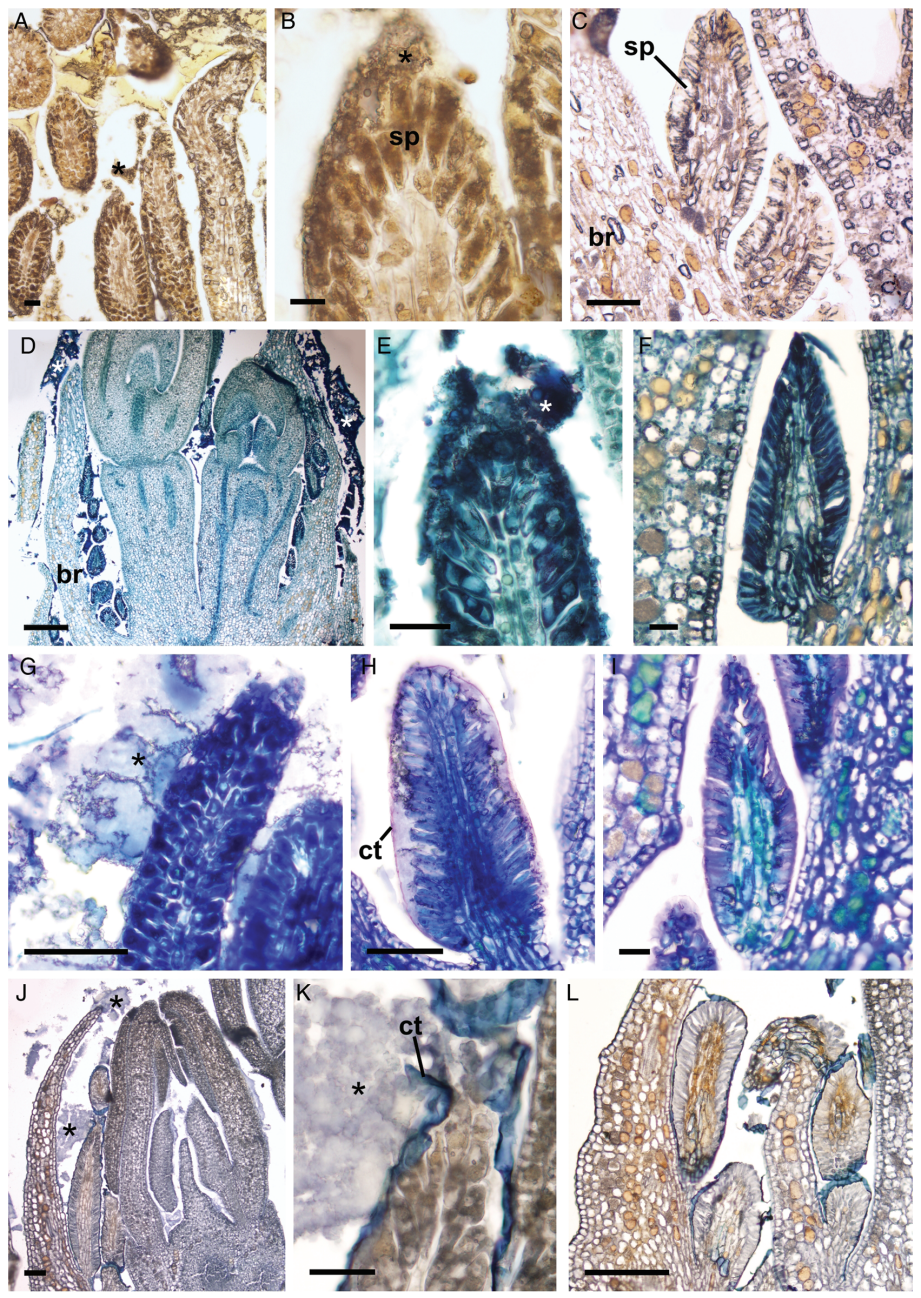
Histochemical characterization of the exudate of colleters. (A, B, D, E, G, H, J and K) Catuaí; (C, F, I and L) Decaffito. (A–C) Wagner's reagent: note the secretory palisade epidermis (sp) and the extracellular exudate (*); (D–F) aniline blue black; (G–I) Nile blue; note in (H) the cuticle (ct) displacement and subcuticular space; (J–L) Sudan black B; note in (K) the cuticular rupture in the apical portion of the colleter, releasing the exudate (*). Scale bars: A, E, F, I and K = 20 µm; B = 10 µm; C, G, H and J = 50 µm; D = 200 µm; L = 100 µm.

Histochemical tests revealed the complex and heterogeneous chemical nature of the exudate detected on the surface of the flower buds and inside the secretory cells of the colleters. The exudate is composed of polysaccharides (Fig. 5E–G), pectic substances (Fig. 5H–J), alkaloids (Fig. 6A and B), proteins (Fig. 6D–F) and lipophilic substances (Fig. 6G–L). The release of the exudate is abundant in Catuaí (Figs 5E, H, I and 6A, B, D, E, G, H, K, L) and scarce in Decaffito (Figs 5F, G, J and 6F, I, L). As expected, alkaloids were not detected in the exudate or inside the secretory cells of the colleters of Decaffito (Fig. 6C).

The exudate released by the secretory epidermal cells between the cuticular layer and the wall layers below led to the formation of large subcuticular spaces in which the exudate accumulated (Fig. 6H). This accumulation creates pressure under the cuticle, causing it to rupture and release the exudate (Figs 3E–G and 6K).

### Ultrastructure of colleters

**Figure 7.**
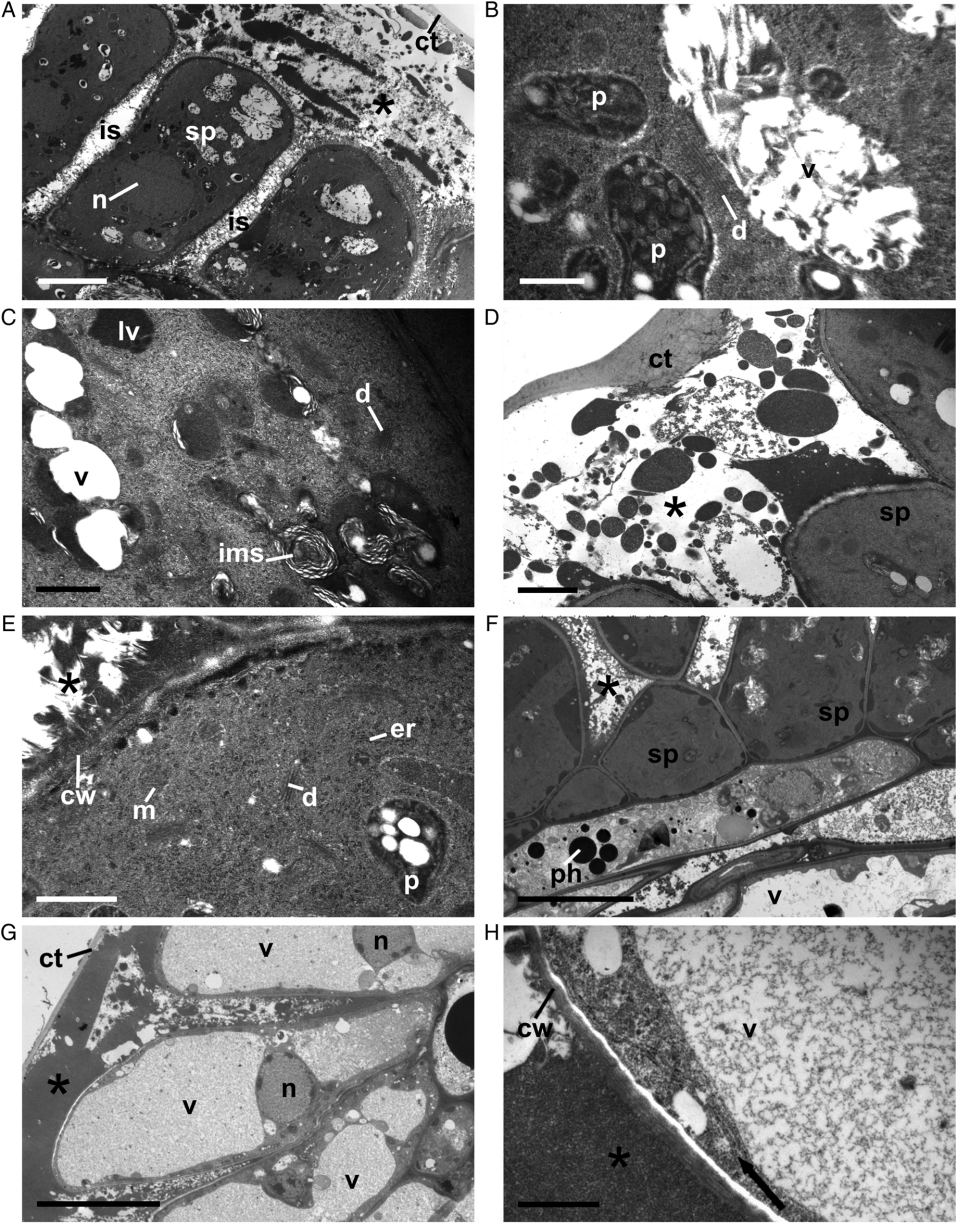
Transmission electron micrographs of Catuaí colleters. (A–F) Colleters in the secretory stage; (G and H) colleters close to senescence. (A) Secretory palisade epidermis (sp): note the dense cytoplasm, large nucleus (n), cuticle (ct) displacement and subcuticular space with exudate (*) and intercellular space (is); (B, C and E) details showing small vacuoles (v), vacuoles with an inner membrane system (ims), dictyosomes (d), plastids (p), lipid vesicles (lv), mitochondria (m) and rough endoplasmic reticulum (er); note in (D) the wide subcuticular space with heterogeneous exudate; note in (F) that the non-secretory parenchyma cell axis accumulates phenolic compounds; (G) secretory palisade epidermis close to senescence: note that the vacuoles increase in size and fuse; (H) the cytoplasm of these secretory cells consists of a thin peripheral layer close to the cell wall (cw): note the endoplasmic reticulum parallel to the plasma membrane (arrows). Scale bars: A = 5 µm; B = 500 nm; C, E and H = 1 µm; D = 2 µm, F and G = 10 µm.

***Catuaí.*** The secretory cells of the colleters of this coffee cultivar have a dense cytoplasm, an evident nucleus, small vacuoles, vesicles containing lipid-like substances, several dictyosomes, mitochondria and rough endoplasmic reticulum (Fig. 7A–E). The plastid matrix is granular with starch grains (clear bodies), classified as amyloplast (Fig. 7B and E). Some small vacuoles have an internal membrane system (Fig. 7C). The exudate is clearly heterogeneous (Fig. 7A, D and G), and it occupies intercellular spaces and the large subcuticular spaces formed by the separation of the cell wall from the cuticle. The non-secretory parenchyma cells of the central axis have a low-density content, and phenols are present (Fig. 7F). In the secretory cells near the senescent phase, the small vacuoles fuse, forming a large vacuole that occupies most of the protoplast interior (Fig. 7G). The cytoplasm becomes restricted to the periphery of the cell, and the rough endoplasmic reticulum appears to be parallel to the plasma membrane (Fig. 7H).

**Figure 8.**
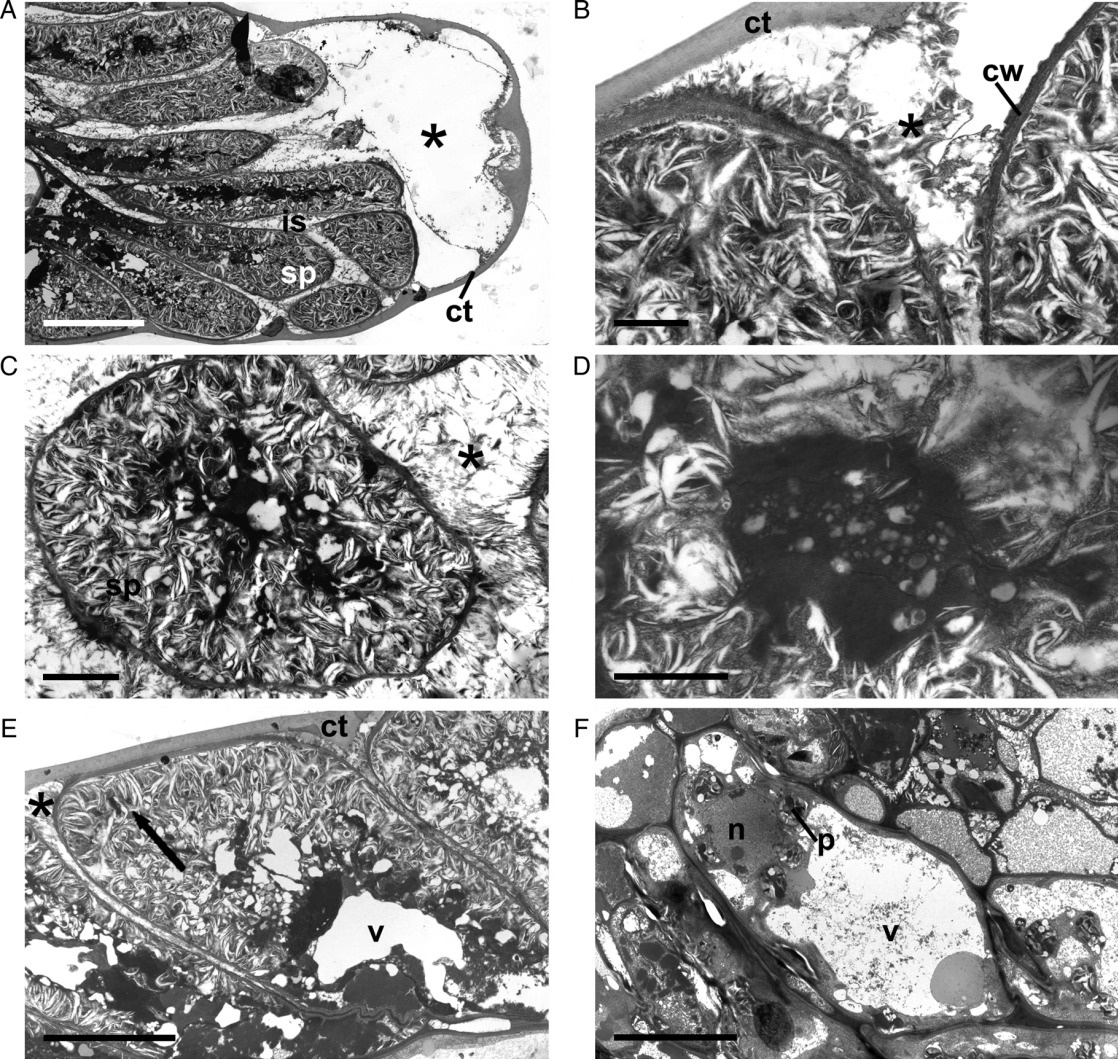
Transmission electron micrographs of Decaffito colleters. (A) Secretory palisade epidermis (sp) in the secretory stage; note the cuticle (ct) displacement and subcuticular space with exudate (*) and intercellular space (is); (B) details of the cell wall (cw) of secretory cells and exudate; (C and D) cross-section of the secretory cell: note the completely disorganized cytoplasm; (D) details of the centre of the secretory cell: note that no intact organelles were observed; only cytoplasm darkening was observed; (E) longitudinal section of the secretory cell: note the exudate inside (arrows) and outside (*) the cell and vacuoles (v); note in (F) the normal non-secretory parenchyma cell axis for the nucleus (n), plastids (p) and large vacuole (v). Scale bars: A and F = 10 µm; B and D = 1 µm; C = 2 µm; E = 5 µm.

***Decaffito.*** The secretory cells of the colleters of Decaffito show marked changes compared with Catuaí as shown by the ultrastructural analysis (Fig. 8A–E). Little exudate is produced, and it seems that it is either not released or when small amounts are released it is still enough to lead to the formation of subcuticular and intercellular spaces (Fig. 8A–C and E). Inside the cell, it is not possible to identify any organelles or even the nucleus, with only a darkened central cytoplasm (Fig. 8A and C–E) and a network of translucent tubule-like structures (Fig. 8C–E) visible. The non-secretory parenchyma cells of the central axis have characteristics similar to those of the Catuaí cell, with low-density content, large vacuoles and a detectable presence of the nucleus and plastids (Fig. 8F).

## Discussion

Stipules protect vegetative buds and leaf primordia (Lubbock 1890; Paiva and Machado 2006*a*, *b*), and bracts protect reproductive structures (Bell 2008). Thus, the presence of colleters in the stipules (Patel and Zaveri 1975) and bracts of *C. arabica* reinforces the idea that they may have a protective role. Colleters occur exclusively on the adaxial face of bracts (Lersten 1974*a*) and are positioned above and adjacent to the reproductive meristem. The beginning of the secretory phase occurs prior to the development of flower organs, and as the flower buds start to develop, they are already covered by the exudate released from the colleters.

Both coffee plants in this study were grown in Campinas (SP, Brazil), where it is well established that coffee floral buds start to differentiate from axillary buds in January at leaf axils that pre-formed in August of the previous year. The formation of flower primordia occurs in March/April (Majerowicz and Söndahl 2005). Starting in May, the temperature and rain precipitation decline and remain low compared with other months until August/September. During this winter period, the vegetative growth of the flower buds ceases, and they remain dormant (Majerowicz and Söndahl 2005). The same climatic conditions apply to Catuaí and Decaffito, although the flower buds of the latter remain dormant, and no growth is observed. Because Decaffito does not have the presumed protection of the colleter exudate, the flower buds are exposed to low air humidity and may dehydrate. We suggest here that the colleter exudate in coffee also functions to keep the petals united, acting as an adhesive, by ‘sealing’ the bud. Once closed, the flowers may be partially protected from dehydration. In Decaffito, the petals are freed due to the lack of exudate. They also lose water from the external surface due to the presence of many stomata (see Fig. 4G), which may cause differential tension between the internal and external cell surfaces, forcing the petals to curve and open precociously (see Fig. 1A) before flower development is complete and when the flowers are still dormant. At the beginning of the rainy season, in September/October, the dormancy is broken and anthesis occurs after 10–12 days of the first rain (Majerowicz and Söndahl 2005). This is observed for both Catuaí and Decaffito, whose flowers are smaller than those of the former, most likely as a consequence of dehydration stress during the dry season (Fig. 1B). Thus, considering that Catuaí and Decaffito flowers differ regarding the presence of exudate, the Decaffito flowers provide functional proof of the role of colleters in protecting coffee flowers from dehydration and controlling their opening.

The histochemical evaluation showed that the difference in composition of the exudate of Catuaí and Decaffito was the absence of alkaloids in the latter, as would be expected given that Decaffito was selected for low caffeine content after sodium azide mutagenesis. The marked detection of caffeine in Catuaí is in agreement with chemical analysis, which showed that the caffeine content in the coffee flowers is among the highest of the different parts of the coffee tree (Hamidi and Wanner 1964; Raju and Gopal 1979).

The histochemical tests also revealed that the exudate composition was highly heterogeneous and complex. Polysaccharides, pectic substances, alkaloids, proteins and lipophilic substances were detected. Complex polysaccharide polymers of high molecular mass seem to play a role as an adhesive to aid in seed dispersion by fixing them to animals and by helping carnivorous plants to capture insects or to lubricate the root apex and facilitate interaction with microorganisms (Fahn 1988 and references therein). Additionally, the hydrophilic characteristics of these polymers seem to assist in maintaining appropriate humidity levels in the meristem and developing organs during dry periods, when soil water content and air humidity are low and temperatures are high (Kronestedt-Robards and Robards 1991; Paiva 2009). On the other hand, the colleter exudate is insoluble in water, which is most likely related to the lipid-like substances, produced to prevent water loss (Thomas and Dave 1989). The Catuaí exudate showed intense colouration for lipids. Kronestedt-Robards and Robards (1991) suggested that these substances could also inhibit the development of pathogenic microorganisms. Similarly, Paiva and Machado (2006*a*) suggested that proteins found in the exudate of colleters of *C. brasiliense* may have an anti-pathogenic function because they found enzyme activities related to chitinases and β-1,3-glucanases, which are usually related to protection against pathogens (Goy *et al*. 1992; Giannakis *et al*. 1998). The presence of protein in the exudate of colleters has also been related to the protection of meristems (Klein *et al*. 2004; Gonzalez and Tarragó 2009) but without a defined function. Although controversial (Guerreiro Filho and Mazzafera 2000, 2003), caffeine has been suggested to protect plants against insect attack (Nathanson 1984). However, it is noteworthy that at any time point, the Decaffito flowers were not observed as more likely to be under attack by insects or microorganisms than Catuaí or any other known *C. arabica* cultivar or coffee species containing caffeine (P. Mazzafera, pers. observ.), which suggests that, at least in coffee, the caffeine in the exudate from colleters does not have a function related to pest or pathogen protection.

The exudate covering the Catuaí flowers is mainly composed of polysaccharides and pectic compounds, which in turn seems to explain the presence of numerous dictyosomes in the secretory cells. Proteins were also densely stained. The secretion of protein–carbohydrate mucilage indicates participation of the Golgi complex and amyloplasts as well as the rough endoplasmic reticulum in the release process (Fahn 1988; Evert 2007). Amyloplasts are abundant in nectariferous tissue (Fahn 1988). They can act as organelles for the storage of substances necessary for the synthesis of the polysaccharide component of the nectar (Rachmilevitz and Fahn 1973; Nepi *et al.* 1996).

Epidermal cells of the colleters of Catuaí showed normal and common characteristics of secretory structures, with an evident nucleus, dense cytoplasm, various dictyosomes and mitochondria. However, the secretory cells of Decaffito did not show any distinguishable organelles, not even the nucleus, but only a darkened cytoplasm. In these cells, the exudate is produced in lower amounts than in Catuaí, and it is not secreted.

The exudate produced in Catuaí and Decaffito accumulates in subcuticular and intercellular spaces. Paiva and Machado (2006*b*) and Appezzato-da-Gloria and Estelita (2000) argued that such subcuticular spaces are formed by dissolution of the middle lamella due to enzyme activities along the anticlinal walls of the epithelial cells. Such processes increase the surface area from which the exudate is released as well as the space in which it can accumulate. Rupture of the cuticle by an increase in pressure caused by exudate accumulation in the subcuticular space has been observed in the colleters of other species of Rubiaceae (Thomas and Dave 1990), Caryocaraceae (Paiva and Machado 2006*a*) and Apocynaceae (Thomas and Dave 1989), which strongly suggests an absence of pores in the cuticle to facilitate exudate release.

Like any other secretory structure, colleters senesce after a secretory phase in which marked anatomical and ultrastructural alterations occur (Dickinson 2000). In the colleters of Catuaí, the main alteration observed with senescence was the state of the cytoplasm, from dense to less dense, and an enlargement of the vacuole. During the senescing phase of the colleters of *B. nicholsonii*, the secretory cells showed a disorganized system of endomembranes, and it was not possible to distinguish organelles, suggesting programmed cell death (Miguel *et al*. 2010). We could not visualize or distinguish any structural organization inside the secretory cells of the colleters of Decaffito in any phase. We speculate that such an occurrence is most likely related to a precocious programmed cell death process.

## Conclusions

The Decaffito plants have very low caffeine content in all tissues, and this characteristic is profoundly associated with precocious flower opening (Borrell 2012; P. Mazzafera, unpubl. res.). Similar to natural mutants of *C. arabica* (Silvarolla *et al.* 2004), Decaffito plants accumulate theobromine, indicating a metabolic blockade of the last step of caffeine biosynthesis (Mazzafera *et al.* 2009). Although it is still not clear what controls caffeine biosynthesis in Decaffito coffee mutants, the associated and undesirable precocious flower opening characteristic provides the first functional proof of the role of colleters and their exudate in protecting flowers against exposure to dry atmospheres and acting as an adhesive to keep the petals united until anthesis. Additionally, although not reported here, we observed a lack of exudate on leaf buds, suggesting that the mutation in Decaffito might also affect the production and release of exudate in the colleters in different plant organs.

## Sources of Funding

This work was financially supported by Fundação de Amparo à Pesquisa do Estado de São Paulo (FAPESP—grants 2005/59775-5 and 2008/54040-5).

## Contributions by the Authors

All the authors contributed to a similar extent overall.

## Conflicts of Interest Statement

None declared.
